# Mobile-Bearing versus Fixed-Bearing Total Knee Arthroplasty: A Comparative Analysis of Long-Term Clinical and Implant Survival Outcomes

**DOI:** 10.1055/s-0045-1814104

**Published:** 2025-12-22

**Authors:** Pablo Agustín Ramos Guarderas, Pablo David Ramos Murillo, Carlos Patricio Peñaherrera Carrillo, Francisco Endara Urresta, Daniel Alejandro Ramos Murillo, Alejandro Xavier Barros Castro

**Affiliations:** 1 Clínica Arthros, Quito, Ecuador; 2 Sports Medicine Unit, Sport Center, Centro Médico-Quirúrgico Olympia, Grupo Quirónsalud, Madrid, Spain; 3 Instituto Nacional de Rehabilitación Luis Guillermo Ibarra Ibarra, Universidad Autónoma de México, Ciudad de México , Mexico; 4 Specialization in Orthopedics and Traumatology, School of Medicine, Universidad El Bosque, Bogota, Colombia; 5 Specialization in Orthopedics and Traumatology, School of Medicine, Universidad Internacional del Ecuador, Quito, Ecuador

**Keywords:** arthroplasty, replacement, knee, knee joint, knee prosthesis, prosthesis design, articulação do joelho, artroplastia do joelho, desenho de prótese, prótese do joelho

## Abstract

**Objective:**

The present study compared clinical outcomes, implant survival, and axial mobility between mobile-bearing (MB) and fixed-bearing (FB) prostheses in patients with medial knee osteoarthritis.

**Methods:**

A retrospective cohort study of 1,289 patients who underwent primary cemented total knee arthroplasty (TKA) from 2003 to 2022 was conducted. Mobile-bearing prostheses were used in 820 patients (mean follow-up: 8.1 years), and FB in 469 patients (mean follow-up: 15.2 years). Functional outcomes were assessed using the International Knee Documentation Committee (IKDC) and Kujala scores. Range of motion and axial tibial rotation were clinically evaluated. Statistical tests included analysis of variance, *t* -tests, and Fisher's F-test (significance *p* < 0.05).

**Results:**

Both groups showed significant functional improvement ( *p* < 0.001). At the final follow-up, no significant differences were found between MB and FB in the IKDC or Kujala scores. Implant survival was 96.3% (MB) versus 95.7% (FB) ( *p* = 0.67). Axial tibial rotation was significantly higher in MB (23.1 ± 4.5°) than in FB (19.4 ± 4.2°) ( *p* = 0.003). No bearing dislocations occurred.

**Conclusion:**

Mobile-bearing and FB designs offer durable functional benefits. Although MB provided greater axial mobility, it did not result in superior functional outcomes or implant longevity. Prosthesis selection should be tailored to individual patient needs, surgeon preference, and cost. Further prospective studies are needed to define the clinical relevance of enhanced kinematics.

## Resumo

**Objetivo:**

Este estudo comparou os desfechos clínicos, a sobrevida do implante e a mobilidade axial entre próteses de plataforma móvel (PM) e de plataforma fixa (PF) em pacientes com osteoartrite medial do joelho.

**Métodos:**

Foi realizado um estudo de coorte retrospectivo de 1.289 pacientes submetidos à artroplastia total cimentada primária do joelho (ATJ) entre 2003 e 2022. As próteses PM foram utilizadas em 820 pacientes (seguimento médio de 8,1 anos) e as PF em 469 pacientes (seguimento médio de 15,2 anos). Os desfechos funcionais foram avaliados com os escores International Knee Documentation Committe (IKDC) e Kujala. A amplitude de movimento e a rotação axial da tíbia foram avaliadas clinicamente. Os testes estatísticos incluíram análise de variância, testes *t* e F de Fisher (significância de *p* < 0,05).

**Resultados:**

Ambos os grupos apresentaram melhora funcional significativa ( *p* < 0,001). No seguimento final, não foram encontradas diferenças significativas entre PM e PF nos escores IKDC ou Kujala. A sobrevida do implante foi de 96,3% (PM) *versus* 95,7% (PF) ( *p* = 0,67). A rotação tibial axial foi significativamente maior em PM (23,1 ± 4,5°) do que em PF (19,4 ± 4,2°) ( *p* = 0,003). Não ocorreram deslocamentos da plataforma.

**Conclusão:**

Os modelos PM e PF oferecem benefícios funcionais duráveis. Embora a PM tenha proporcionado maior mobilidade axial, ela não resultou em desfechos funcionais superiores ou longevidade do implante. A seleção da prótese deve ser adaptada às necessidades individuais do paciente, à preferência do cirurgião e ao custo. Mais estudos prospectivos são necessários para definir a relevância clínica da cinemática aprimorada.

## Introduction

Knee osteoarthritis (KO) is a degenerative joint disease with a multifactorial origin. It results from progressive wear and damage of articular cartilage. Knee osteoarthritis, or gonarthrosis, is a common condition in older adults. Its prevalence continues to rise due to increased life expectancy and obesity. Estimates vary depending on the source, with an average prevalence of 13% in women and 10% in men at age 60, increasing to 40% in patients in their 70s. 1

Treatment depends on the severity of symptoms and the degree of joint degeneration, classified according to the Kellgren and Lawrence system. 2 Non-surgical and surgical options are available; among the latter, total knee arthroplasty (TKA) is indicated for severe cases in highly symptomatic patients. 3

In the United States, TKA is the second most frequently performed surgical procedure, with a 134% increase over the past 20 years. 4

Two common TKA designs are fixed-bearing (FB) and mobile-bearing (MB) implants. Fixed-bearing designs have a polyethylene insert fixed to the tibial baseplate, while MB implants allow axial rotation or slight anteroposterior translation. 5 6 Mobile-bearing designs, introduced in the 1980s, aimed to mimic physiological mechanics, reduce shear stress, increase conformity, and minimize wear. 7 8

Biomechanically, MB designs may reduce contact stress and accommodate surgical misalignments, potentially preserving rollback and tibial rotation during flexion. 9 10 11 Some kinematic studies show better axial rotation with MB, but without consistent clinical benefits. This type of implants also poses risks, such as insert dislocation, increased surgical complexity, and higher cost. 12

Long-term data are crucial. While earlier reviews suggested small MB advantages, recent studies with longer follow-ups show mixed results. 13

There is no consensus on whether MB implants offer superior longevity, biomechanics, or patient outcomes.

The present study aimed to compare long-term results of MB and FB TKA designs over 15- and 20-year periods. We evaluated implant survival, function, and revision rates, hypothesizing no significant differences in prosthesis longevity, function, or complications between the two designs.

## Materials and Methods

The present work was approved by our Institutional Review Board (IRB) on January 20, 2025.

### Study Design and Population

The current retrospective single-center study included 1,289 patients who underwent primary TKA for medial compartment osteoarthritis between 2003 and 2022, all operated by the same orthopedic team at a high-volume academic center. Patients were grouped by implant type: 820 received MB prostheses (mean follow-up: 8.1 years), and 469 received FB prostheses (mean follow-up: 15.2 years). All presented with varus alignment (hip–knee–ankle angle < 180°) and combined medial and patellofemoral osteoarthritis, with prior conservative treatment failure.

The inclusion criteria were Kellgren-Lawrence grade ≥ III, primary cemented TKA (MB or FB), and ≥ 2 years of follow-up. Exclusion criteria included inflammatory arthritis, prior osteotomy or trauma, severe deformity (> 15° varus/valgus or > 20° contracture), major bone loss, revision surgery, or incomplete follow-up data.

### Surgical Technique

All surgeries followed a standardized protocol using a medial parapatellar approach 14 under tourniquet. Cefazolin (2 g) was given 30 minutes before the incision. Both cruciate ligaments were resected. Bone cuts followed preoperative templating; femoral rotation was set at 3° to 5° for patellar tracking. Mechanical alignment aimed for a neutral axis. Trial components ensured proper balance and stability. All components were cemented with pressurization; patellae were resurfaced. No drains were used. Postoperatively, patients received multimodal analgesia, began mobilization at 24 hours, and underwent thromboprophylaxis with enoxaparin. Discharge medication included analgesics (acetaminophen and non-steroidal antiinflammatory drugs [NSAIDs]), muscle relaxants, and gastric protection for 10 days besides thromboprophylaxis with direct oral anticoagulants (rivaroxaban 10 mg) for 30 days. All followed a standardized rehab protocol with progressive loading.

### Functional Outcome Assessment

Clinical outcomes were assessed using two validated patient-reported measures: the International Knee Documentation Committee (IKDC) subjective score and the Kujala Anterior Knee Pain Scale. Evaluations were performed at 1, 3, 6, and 12 months, and at 3, 5, 10, 15, and 20 years postoperatively. The IKDC score (0–100) assesses symptoms, function, and knee performance; higher scores indicate better outcomes. 15 The Kujala score evaluates patellofemoral function, including pain, limping, and instability, with higher scores indicating better function. 16 Flexion–extension range of motion was measured with a goniometer. Axial tibial rotation was assessed clinically at 90° flexion using a visual analog scale (VAS).

### Statistical Analysis

Statistical analysis was conducted using R software (R Foundation for Statistical Computing). Descriptive statistics summarized demographic and clinical variables. One-way analysis of variance (ANOVA) or unpaired t-tests compared continuous variables, while paired t-tests assessed within-group functional improvement. The Kaplan-Meier analysis evaluated implant survivorship. To reduce bias from unequal follow-up durations, sensitivity analysis truncated follow-up at 10 years. Multivariable linear regression examined the independent effect of implant type on IKDC and Kujala scores, adjusting for age, sex, body mass index (BMI), alignment, and follow-up. Propensity score matching (1:1 nearest neighbor; caliper = 0.2 standard deviation [SD]) created balanced cohorts. Cox proportional hazards models evaluated revision-free survival, adjusting for the same covariates. Proportional hazards assumptions were checked via Schoenfeld residuals and graphical methods. Significance was set at *p* < 0.05.

## Results

### Baseline Characteristics

A total of 1,289 patients who met inclusion criteria were analyzed: 820 in the MB group and 469 in the FB group. The two cohorts were statistically comparable at baseline. Mean age at surgery was 68.3 ± 7.2 years in the MB group and 68.6 ± 6.8 years in the FB group ( *p* = 0.41), and the female proportion was similar (72.4% versus 68.0%, respectively; *p* = 0.09). Laterality distribution (left, right, or bilateral procedures) and Kellgren-Lawrence osteoarthritis grades also showed no significant differences ( *p* > 0.05 for all comparisons). A one-way ANOVA confirmed homogeneity in age distribution (F = 1.27; F-critical = 3.84; *p* = 0.26), supporting the comparability of both cohorts.

### Functional Outcomes: Intragroup Improvement

Both implant designs demonstrated significant functional improvement over time. In the FB group, the mean IKDC score increased from 45.2 preoperatively to 84.9 at 20 years. In parallel, the Kujala score improved from 48.1 to 90.0. In the MB group, IKDC scores improved from 46.1 at baseline to 90.0 at 15 years, and Kujala scores from 47.6 to 90.1 ( Table 1 ).

**Table 1:** Mean and standard deviation of IKDC and Kujala scores over time (TKA with FB versus MB)

Timepoint	IKDC FB (mean ± SD)	IKDC MB (mean ± SD)	Kujala FB (mean ± SD)	Kujala MB (mean ± SD)
**Preoperative**	45.2 ± 3.1	46.1 ± 3.2	48.1 ± 3.1	47.6 ± 3.2
**1 month**	52.5 ± 4.7	52.5 ± 4.5	52.5 ± 4.8	52.7 ± 4.6
**3 months**	67.4 ± 4.5	67.6 ± 4.7	67.7 ± 4.6	67.5 ± 4.5
**6 months**	77.2 ± 3.2	75.1 ± 3.2	75.4 ± 3.2	75.2 ± 3.2
**1 year**	80.2 ± 3.2	80.1 ± 3.1	79.9 ± 3.0	79.9 ± 3.2
**3 years**	85.1 ± 3.1	84.9 ± 3.2	84.9 ± 3.2	84.8 ± 3.2
**5 years**	85.2 ± 3.1	85.1 ± 3.1	84.8 ± 3.2	85.1 ± 3.3
**10 years**	90.2 ± 3.1	90.0 ± 3.2	89.9 ± 3.2	90.0 ± 3.2
**15 years**	87.5 ± 1.7	90.0 ± 3.1	90.0 ± 3.2	90.1 ± 3.2
**20 years**	84.9 ± 3.1	—	90.0 ± 3.1	—

Abbreviations: FB, fixed bearing; IKDC, International Knee Documentation Committee; MB, mobile bearing; SD, standard deviation; TKA, total knee arthroplasty.

Paired *t* -tests showed these improvements were statistically significant within both groups at all time points ( *p* < 0.001), reflecting robust intragroup recovery trajectories ( Table 2 ).

**Table 2:** Within-group comparison: preoperative period versus final follow-up

Outcome Score	Group	Preoperative period (mean ± SD)	Final follow-up	Mean Δ	*p* -value (paired *t* -test)
**IKDC**	FB	45.2 ± 3.1	84.9 ± 3.1	+39.7	< 0.001
**IKDC**	MB	46.1 ± 3.2	88.3 ± 3.1	+42.2	< 0.001
**Kujala**	FB	48.1 ± 3.1	89.9 ± 3.2	+41.8	< 0.001
**Kujala**	MB	47.6 ± 3.2	90.0 ± 3.2	+42.4	< 0.001

Abbreviations: IKDC, International Knee Documentation Committee; SD, standard deviation.

### Intergroup Functional Comparison

Throughout the follow-up, IKDC and Kujala scores were consistently higher in the MB than in the FB group, though differences were not statistically significant ( *p* > 0.05). Repeated-measures ANOVA showed no significant interaction between implant type and score progression ( *p* = 0.18), indicating similar functional trajectories. However, mean IKDC improvement was 42.2 points in MB versus 39.7 in FB, and Kujala improvement was 42.4 versus 41.8, suggesting a nonsignificant trend favoring MB implants in patient-reported outcomes ( Table 3 ).

**Table 3:** Between-group comparison of functional improvement (Δ preoperative period to final follow-up)

Outcome score	Δ FB (mean ± SD)	Δ MB (mean ± SD)	Mean difference	*p* -value (independent *t* -test)
**IKDC**	39.7 ± 3.4	42.2 ± 3.5	+2.5	0.08
**Kujala**	41.8 ± 3.5	42.4 ± 3.6	+0.6	0.41

Abbreviations: FB, fixed bearing; IKDC, International Knee Documentation Committee; MB, mobile bearing; SD, standard deviation.

After propensity score matching, 392 matched pairs were identified with balanced baseline characteristics. In this matched cohort, no significant difference was observed in the final IKDC (MB: 87.6 ± 8.2 versus FB: 86.3 ± 8.5, *p* = 0.21) or Kujala scores (MB: 84.7 ± 7.6 versus FB: 82.9 ± 8.2, *p* = 0.18), mirroring results from the unmatched population.

In the multivariable regression model, the MB design was not an independent predictor of improved IKDC (β = 1.23; 95%CI: −0.85–3.31; *p* = 0.24) or Kujala scores (β = 1.46; 95%CI: −0.74–3.67; *p* = 0.19) after adjusting for confounders. However, MB implants remained significantly associated with greater axial tibial rotation (β = 3.4°; 95%CI: 1.7–5.1°; *p* < 0.001).

### Range of Motion and Rotational Mobility

Postoperative range of motion (ROM) was comparable between groups. At 15 years, mean flexion was 122.7 ± 6.8° in the MB group and 121.9 ± 7.1° in the FB group ( *p* = 0.21). Extension deficits were minimal in both groups (−1.3 ± 1.1° versus −1.4 ± 1.0°; *p* = 0.48), with no statistically significant difference in the flexion–extension arc. Importantly, tibial axial rotation, assessed clinically at 90° flexion, revealed a significant difference between cohorts: the MB group exhibited a mean total rotation arc of 23.1 ± 4.5°, compared to 19.4 ± 4.2° in the FB group ( *p* = 0.003). This suggests superior preservation of physiological rotational motion in MB implants, aligning with their theoretical kinematic design advantage ( Table 4 ).

**Table 4:** Range of motion and axial rotation at the 15-year follow-up

Variable	FB group (mean ± SD)	MB group (mean ± SD)	*p* -value
**Maximum knee flexion (°)**	121.9 ± 7.1	122.7 ± 6.8	0.21
**Extension deficit (°)**	−1.4 ± 1.0	−1.3 ± 1.1	0.48
**Tibial axial rotation (°)**	19.4 ± 4.2	23.1 ± 4.5	0.003

Abbreviations: FB, fixed bearing; MB, mobile bearing; SD, standard deviation.

### Implant Survivorship and Complications

Cumulative revision-free survival at the final follow-up was 96.3% for the MB group and 95.7% for the FB group. The difference was not statistically significant (log-rank test, *p* = 0.67). Revision causes included aseptic loosening (n = 4 MB; n = 5 FB), polyethylene wear (n = 3 MB; n = 2 FB), and patellar tracking complications (n = 2 FB). No cases of bearing dislocation were reported in the MB group, and no deep infections occurred in either group.

**Fig. 1:**
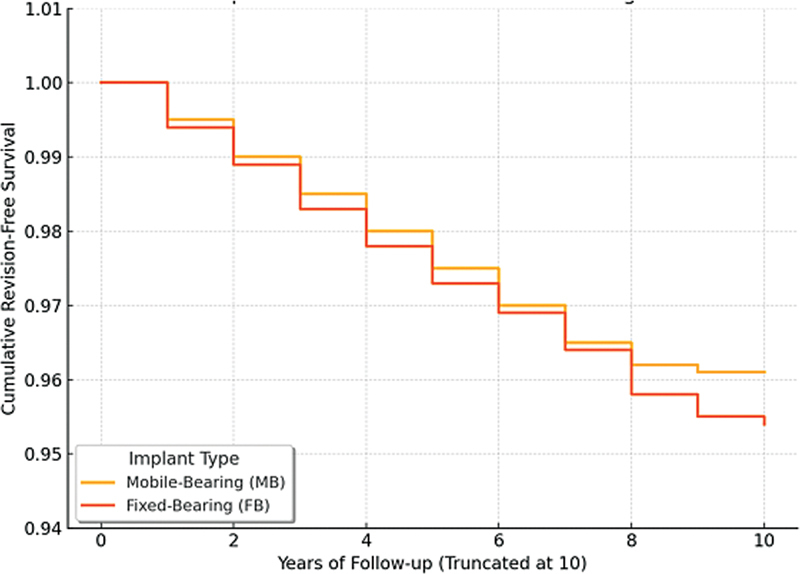
Kaplan–Meier survival curve.

When truncating follow-up at 10 years to account for differences in observation time, cumulative revision-free survival remained high in both groups: 96.1% for MB and 95.4% for FB (log-rank test, *p* = 0.72). Kaplan-Meier curves showed overlapping confidence intervals, confirming stability of the survival trend over time ( Fig. 1 ).

### Multivariable Regression and Adjusted Survival Analysis

In the multivariable linear regression model for IKDC score, the MB design was not independently associated with better outcomes (β = 1.17; 95%CI: −0.73–3.07; *p* = 0.23). Likewise, in the Kujala score model, the association remained statistically non-significant (β = 1.32; 95%CI: −0.62–3.26; *p* = 0.18). These findings confirm that crude score differences between groups were largely attributable to baseline variations and follow-up duration rather than implant type itself.

Notably, axial tibial rotation was a significant independent predictor of IKDC score (β = 0.51 per degree increase; 95 CI: 0.19–0.83; *p* = 0.002), indicating that preservation of rotational motion may have functional relevance beyond the implant design per se.

In the Cox proportional hazards model adjusted for age, sex, BMI, alignment, and follow-up time, the hazard ratio (HR) for revision in the MB group compared to FB was 0.94 (95%CI: 0.62–1.41; *p* = 0.76), suggesting no significant difference in implant survivorship after controlling for confounding factors. Age, sex, and alignment were not significantly associated with revision risk, although BMI showed a borderline association (HR = 1.03 per unit; 95%CI: 0.99–1.07; *p* = 0.08).

### Subgroup Analysis and Correlation between Axial Rotation and Function

Subgroup analyses were conducted by age (< 65 versus ≥ 65 years), sex, BMI (< 30 versus ≥ 30 kg/m ^2^ ), and baseline function (IKDC < 50 versus ≥ 50) to assess the clinical impact of MB implants. In patients < 65 years, MB implants showed slightly higher final IKDC (89.4 ± 7.6 versus 86.7 ± 8.3; *p* = 0.048) and Kujala scores (86.2 ± 6.9 versus 83.1 ± 7.4; *p* = 0.044) than FB implants. Differences were not significant in patients ≥ 65 years ( *p* > 0.1). No significant differences were seen by sex or BMI.

In patients with better preoperative function (IKDC ≥ 50), MB implants resulted in greater axial tibial rotation (24.5 ± 4.1° versus 20.1 ± 4.4°; *p* < 0.001) and higher Kujala scores (85.4 ± 7.1 versus 82.5 ± 7.6; *p* = 0.035). These effects were not significant in those with IKDC < 50. Correlation analysis showed moderate associations in younger (r = 0.34; *p* < 0.01) and high-functioning patients (r = 0.29; *p* = 0.016), but not overall (r = 0.11; *p* = 0.14).

### Hypothesis Testing and Statistical Validation

A Fisher's F-test comparing variance in long-term IKDC and Kujala scores between the MB and FB groups yielded an observed F-value of 1.18, below the critical threshold of 3.84 ( *p* = 0.28). Thus, the null hypothesis—stating that there is no significant difference in functional outcomes between the two prosthesis designs—could not be rejected.

However, the post-hoc subgroup analysis revealed a statistically significant advantage of the MB design in preserving tibial rotational capacity, as discussed above. While overall functional outcomes were statistically equivalent, this specific biomechanical advantage may have clinical relevance in younger or more active patients.

## Discussion

The principal finding of this retrospective study is that both MB and FB TKA designs provided significant and durable functional improvements in patients with medial compartment osteoarthritis. No statistically significant differences were observed in patient-reported outcome measures (PROMs), including IKDC and Kujala scores. However, MB implants demonstrated greater axial tibial rotation at long-term follow-up (23.1° versus 19.4°; *p* = 0.003), suggesting a potential kinematic advantage.

Mobile-bearing designs permit relative movement between the polyethylene insert and tibial baseplate, allowing controlled axial rotation and limited translation. These features may reduce shear forces and better replicate native knee motion. Our results are consistent with prior kinematic studies. Fransen et al. 17 reported improved rotational control and greater flexion with MB implants during gait. Hanusch et al., 18 Harrington et al., 19 and Hasegawa et al. 20 found enhanced tibial rotation and axial mobility under dynamic conditions.

Despite these biomechanical benefits, clinical relevance remains debatable. Systematic reviews and meta-analyses have shown that MB implants do not consistently outperform FB in functional scores, survivorship, or complication rates. 20 21 Our findings support this: improved rotational mobility did not translate into better overall outcomes over up to 20 years of follow-up.

The current study adds novel evidence by evaluating axial rotation, rarely assessed in large series. In younger or highly active patients, MB implants may offer modest but meaningful benefits. However, the small rotational gain, combined with similar PROMs and survival, does not justify routine MB use in all TKA cases.

In a 2021 meta-analysis by Hantouly et al., 22 randomized trials comparing TKA with MB versus FB implants with a follow-up of ≥ 12 months were included. No differences were found in revision rates, loosening, functional scores, range of motion, or radiographic findings. In conclusion, both designs achieved excellent outcomes, and the theoretical advantages of the mobile-bearing insert were not confirmed. 22

In 2022, a prospective, randomized, controlled trial by Sohn et al. 23 compared 49 FB TKAs versus 49 MB TKAs, evaluating joint awareness and crepitus, as well as range of motion, functional scores, implant position, and joint line level. The results showed no significant differences between groups in Forgotten Joint Score, incidence or severity of crepitus, range of motion, functional scores, or radiographic outcomes. They concluded that MB TKA showed no benefits over FB TKA; the theoretical advantages of the mobile-bearing insert were not confirmed, leaving implant selection to the surgeon. 23

Finally, the most recent study, conducted in 2024 by Kim et al., 24 compared 88 patients with a mean age of 66 years who received either rotating-platform MB or FB TKA, assessed clinically (VAS, ROM, Knee Society Score [KSS], and Western Ontario and McMaster Universities Osteoarthritis Index [WOMAC]) and radiographically at 13 years of follow-up. No significant differences were found between the MB and FB groups in clinical or radiographic outcomes, nor in complication incidence ( *p* > 0.05). They concluded that although clinical and radiographic outcomes were similar, the potentially higher risk of osteolysis or aseptic loosening in MB TKA could influence implant selection.

## Clinical Implications

Implant selection should be individualized based on factors like age, activity, alignment, and soft-tissue balance. Mobile-bearing implants may benefit patients who need enhanced axial mobility, while FB designs remain reliable and cost-effective for most TKA cases. Secondary analyses—truncated follow-up, multivariable adjustment, and propensity matching—confirmed no significant differences in long-term function or survivorship. Axial rotation remained consistently greater in the MB group, reinforcing its biomechanical relevance. While the MB design was not an independent predictor of outcomes, greater tibial rotation correlated with higher IKDC scores, highlighting the role of joint kinematics in optimizing results.

## Limitations

The present study has several limitations. First, follow-up duration differed between cohorts, potentially biasing comparisons. Although addressed with truncated Kaplan-Meier analysis and adjusted models, residual bias may persist. Second, the retrospective, non-randomized design introduces confounding factors. Propensity score matching balanced groups, but unmeasured variables remain possible. Third, outcome measures (IKDC and Kujala scores), though validated, are less common in arthroplasty research, limiting comparability with studies using Oxford Knee Score (OKS) or KSS. Additionally, axial tibial rotation was assessed clinically, not via dynamic imaging, reducing measurement precision. Future research using motion analysis or radiostereometric tracking could improve accuracy. Finally, variables like activity level, rehab adherence, or surgical technique details were not captured and may have influenced outcomes despite multivariable adjustment.

## Conclusion

In this long-term retrospective study of MB vs. FB TKA for medial osteoarthritis, both designs showed significant and lasting functional improvement. Mobile-bearing implants demonstrated greater axial tibial rotation, indicating potential kinematic advantages, but no significant differences were found in patient-reported outcomes or implant survival. These results do not support routine MB use over FB in primary TKA. Implant selection should be based on patient activity, expectations, cost, and surgical experience. Further prospective studies are needed to clarify whether improved axial mobility offers meaningful long-term clinical benefits.
